# Silica Nanoparticles Inhibit Responses to ATP in Human Airway Epithelial 16HBE Cells

**DOI:** 10.3390/ijms221810173

**Published:** 2021-09-21

**Authors:** Alina Milici, Alicia Sanchez, Karel Talavera

**Affiliations:** Laboratory of Ion Channel Research, Department of Cellular and Molecular Medicine, VIB Center for Brain & Disease Research, Herestraat 49, 3000 Leuven, Belgium; alina.milici@kuleuven.be (A.M.); alyciasl@gmail.com (A.S.)

**Keywords:** silica, nanoparticles, ATP, purinergic receptor, airway, epithelial cell, intracellular Ca^2+^

## Abstract

Because of their low cost and easy production, silica nanoparticles (SiNPs) are widely used in multiple manufacturing applications as anti-caking, densifying and hydrophobic agents. However, this has increased the exposure levels of the general population and has raised concerns about the toxicity of this nanomaterial. SiNPs affect the function of the airway epithelium, but the biochemical pathways targeted by these particles remain largely unknown. Here we investigated the effects of SiNPs on the responses of 16HBE14o- cultured human bronchial epithelial (16HBE) cells to the damage-associated molecular pattern ATP, using fluorometric measurements of intracellular Ca^2+^ concentration. Upon stimulation with extracellular ATP, these cells displayed a concentration-dependent increase in intracellular Ca^2+^, which was mediated by release from intracellular stores. SiNPs inhibited the Ca^2+^ responses to ATP within minutes of application and at low micromolar concentrations, which are significantly faster and more potent than those previously reported for the induction of cellular toxicity and pro-inflammatory responses. SiNPs-induced inhibition is independent from the increase in intracellular Ca^2+^ they produce, is largely irreversible and occurs via a non-competitive mechanism. These findings suggest that SiNPs reduce the ability of airway epithelial cells to mount ATP-dependent protective responses.

## 1. Introduction

Nanoparticles (NPs) designate small-sized natural or engineered particulate matter with dimensions of less than 100 nm in at least one dimension and physical and chemical properties that differ from those of the bulk material. They are composed of a wide range of materials, such as metals, inorganic carbon, and organic compounds. In the last decades, NPs have gained popularity in many industries, as their unique properties make them suitable for use in cosmetics, biotechnology, food, pharmaceutical and chemical industries [1,2,3,4,5,6,7].

Human exposure to NPs has increased considerably since the nano-technological revolution. Their small dimensions and unique physico-chemical properties facilitate the ability of nanoparticles to cross different barriers and to reach distal organs [8,9,10,11,12]. They can penetrate the plasma membrane and deposit into subcellular structures such as mitochondria, endoplasmic reticulum and lysosomes, thereby posing potential health threats [13,14,15,16,17]. This, together with their extensive use in consumer products, has raised concerns about the safety of NPs [8,18]. A growing body of evidence points towards the deleterious effects of NPs [19,20,21,22]. However, not all NPs are harmful, and some of them are engineered to be used in medicine as drug nanocarriers due to their specific interaction with target organs [6,23]. NPs can enter our bodies by inhalation, ingestion, injection or through the skin, but the main entry route is the airway epithelium [8,24].

Besides its role in gas exchange, the airway epithelium protects the body from foreign substances. The cells lining the respiratory tract have key roles in mucociliary clearance and contribute to the initiation of airway inflammation [25,26,27,28]. In the airways, NPs may interact with epithelial cells and alter their exocrine and paracrine functions, and with sensory neurons, which initiate the cough reflex and neurogenic inflammation [29]. It has been shown that upon inhalation, NPs impair the normal functioning of the airways by inducing inflammation, increasing the secretion of mucus and inhibiting the ciliary beat frequency [30,31,32,33,34].

Silica particles (SiNPs), the subject of this study, are among the most produced synthetic nanomaterials worldwide due to their tunable physico-chemical properties, stability, low cost and easy production [7,35,36]. SiNPs have been shown to induce lung inflammation, cytotoxic responses such as apoptosis, DNA damage and endoplasmic reticulum stress; they increase the reactive oxygen species (ROS) production by altering Ca^2+^ homeostasis, disrupt the plasma membrane upon uptake and alter membrane fluidity and integrity [7,10,15,20,21,37,38]. Previous studies indicate that the effects induced by SiNPs are size- and concentration-dependent and differ across cell types. More pronounced toxic effects have been reported for smaller particles, increased doses and prolonged exposure times [7,15,39]. Very recent studies using three-dimensional model airway and lung preparations have shown that SiNPs induce genotoxicity [40], asymmetric changes in pro-inflammatory cytokine and chemokine expression across the airway barrier and the increase in adhesion molecules in the apical compartment [41].

Despite the extensive research on the toxicity of SiNPs, the mechanisms underlying their deleterious effects are poorly understood. For instance, most of the studies related to SiNPs are focused on chronic effects for in vitro exposure times ranging from several hours up to days [42]. On the other hand, recent research has illustrated the importance of studying also the acute interaction of SiNPs with specific cellular structures and signaling pathways. For instance, SiNP-induced modulation in the time scales of seconds and minutes have been reported for cation-permeable channels that play crucial roles in cell signaling [30,37,39,43,44]. In this sense, ATP-mediated signaling is considered to be involved in inflammatory responses to SiNPs in multiple cell types [45,46,47,48,49,50].

ATP receptors are ligand-gated cation channels (P2X, ionotropic) or G protein-coupled receptors (P2Y, metabotropic) [51,52,53,54]. Activation of P2X receptors leads to extracellular Ca^2+^ influx, resulting in plasma membrane depolarization and direct increase in intracellular [Ca^2+^]. In contrast, activation of P2Y receptors activates inositol triphosphate (IP_3_)-mediated signaling, which opens IP_3_ receptor Ca^2+^ channels in the membrane of endoplasmic reticulum (ER), leading to Ca^2+^ release from these stores [52,55]. In the airways, extracellular ATP signals tissue damage induced by particulates or pathogens, and is therefore considered as a damage-associated molecular pattern (DAMP) [56]. ATP receptors are expressed along the respiratory tract in epithelial cells and in sensory nerves in upper (trigeminal ganglia) and lower airways (nodose and dorsal root ganglia) [57,58,59]. It has been reported that these types of cells express P2X, as well as P2Y receptors. However, airway injury induced by particulate matter inhalation has been predominantly associated with P2Y-mediated signaling in epithelial cells leading to enhanced mucociliary clearance. On the other hand, P2X receptors have a prominent role in sensory nerves, as their activation triggers defensive responses such as cough [56,60,61,62,63,64,65]. Most of the studies addressing ATP-mediated signaling are related to ATP release upon cell damage [66].

The purpose of this study was to determine whether SiNPs affect the responses of airway epithelial cells to ATP. Using cultured human bronchial epithelial (16HBE) cells as a model, we found that acute extracellular application of commercially available SINPs potently reduces the responses to ATP in a concentration-dependent manner. Our results unveil the ATP signaling pathway as a direct cellular target of SiNPs. In the broad context, this suggests that SiNPs affect the ability of airway epithelial cells to respond to environmental and endogenous stimuli that require ATP-mediated signaling.

## 2. Results

### 2.1. Intracellular Ca^2+^ Response of 16HBE Cells to Extracellular ATP

**Figure 1:**
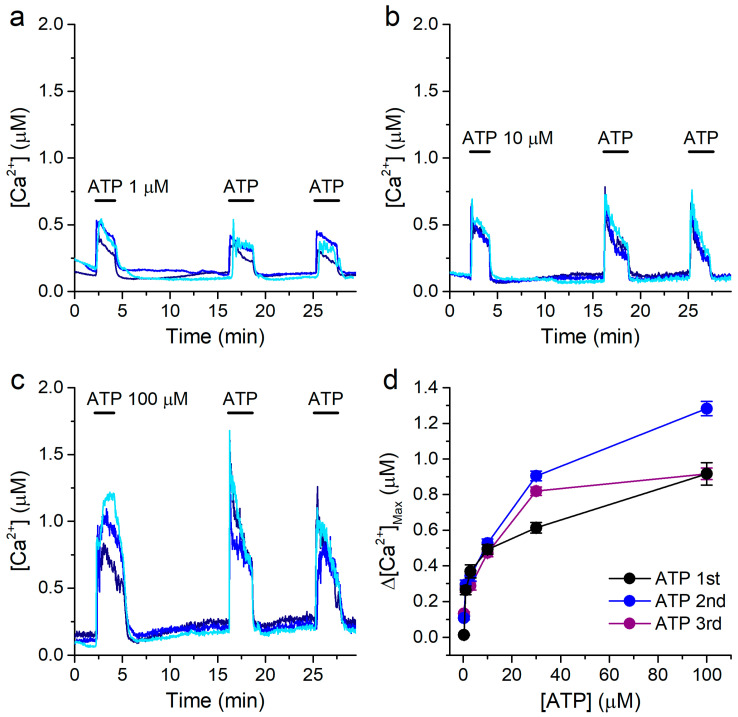
Extracellular ATP induces concentration-dependent responses in 16HBE cells. (**a**–**c**) Examples of intracellular [Ca^2+^] traces showing the responses to extracellular ATP applied at 1 μM (**a**), 10 μM (**b**) or 100 μM (**c**), using a standard Krebs solution. (**d**) Concentration dependence of the amplitude of the responses to the 1st (black), 2nd (blue) and 3rd (purple) applications of ATP. The data are represented as mean ± s.e.m. (*n* = 179, 59, 75, 96, 278 and 128, for ATP 0.3, 1, 3, 10, 30 and 100 μM, respectively).

We first characterized the response of 16HBE cells to extracellular application of ATP, under constant perfusion of standard Krebs solution (containing 1.5 mM Ca^2+^, see Materials and Methods). We determined the amplitude of the increase in intracellular Ca^2+^ concentrations induced by three consecutive applications of ATP, at various concentrations (from 0.3 to 100 μM; Figure 1a–c). The responses very commonly showed a sharp initial upstroke phase, followed by a slow decrease in [Ca^2+^], typical of the activation of ATP receptors [67]. As expected, the response in intracellular [Ca^2+^] was concentration-dependent (Figure 1d).

This type of stimulation protocol allowed us to evoke consecutive responses to ATP in a reliable manner, with no desensitization. Thus, we could use a variant of this protocol to determine if SiNPs affect the responses to ATP, by comparing the amplitudes of the responses to ATP in the absence and in the presence of SiNPs in the same cells.

### 2.2. The Ca^2+^ Responses to ATP Are Mediated by Release from Intracellular Stores

**Figure 2:**
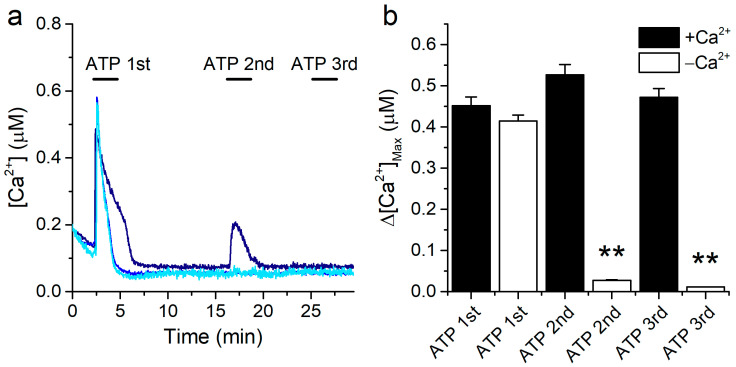
ATP mobilizes Ca^2+^ from intracellular stores. (**a**) Examples of traces of [Ca^2+^] recorded in 16HBE cells showing the effects of repetitive extracellular application of 10 μM ATP in the absence of extracellular Ca^2+^. (**b**) Amplitude of the responses to the 1st, 2nd and 3rd ATP applications in the presence and in the absence of extracellular Ca^2+^. The data are represented as mean ± s.e.m. (*n* = 207, 482, 97, 249, 97 and 247 for the columns from left to right). The asterisks (**) denote statistically significant difference of the data obtained in the Ca^2+^-free condition (−Ca^2+^) with respect to the corresponding control data (+Ca^2+^) (*p* < 0.01; Kolmogorov-Smirnov test).

The intracellular Ca^2+^ responses to ATP may be mediated by metabotropic (P2Y) and/or by ionotropic (P2X) receptors. To determine the origin of the Ca^2+^ increase upon ATP application in our experimental conditions, we performed measurements in the absence of extracellular Ca^2+^. For this, cells were allowed to stabilize in the standard Krebs solution before the experiments and then perfused with a Ca^2+^-free extracellular solution (see Materials and Methods). In the latter condition, a first application of 10 μM ATP elicited sizeable intracellular Ca^2+^ responses, whose amplitudes were not significantly different from the amplitudes of the responses recorded in the presence of extracellular Ca^2+^ (Figure 2a,b, columns ATP 1st). In contrast, the second ATP application induced a transient increase in intracellular [Ca^2+^] only in a very limited number of cells (Figure 2a,b, columns ATP 2nd), and a third application failed to trigger any response (Figure 2a,b, columns ATP 3rd).

These results show that 16HBE cells can support full-sized responses at least to a first application of ATP in the absence of extracellular Ca^2+^. This demonstrates that the responses are mediated by release from intracellular stores.

### 2.3. Concentration-Dependent Inhibition of Responses to ATP by SiNPs

**Figure 3:**
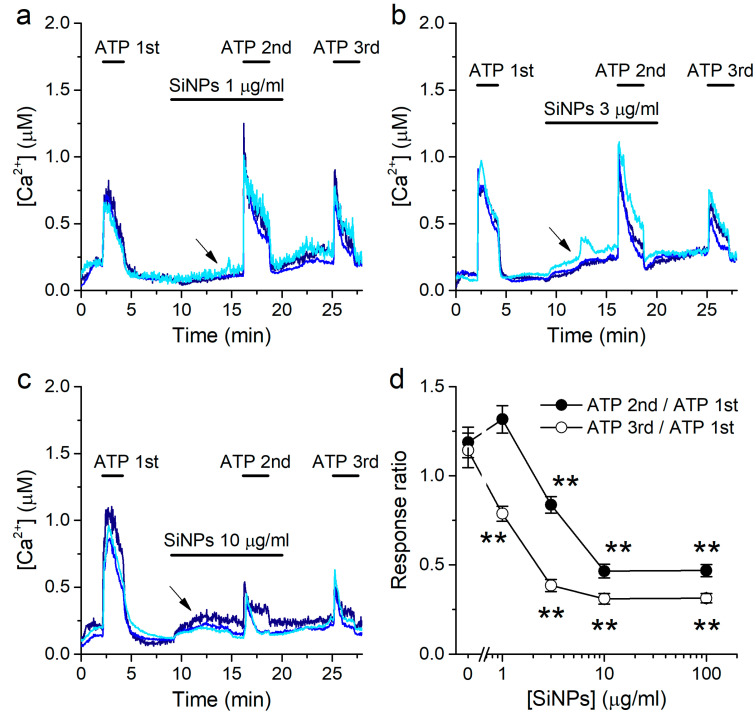
SiNPs induce a concentration-dependent inhibition of the response to ATP. (**a**–**c**) Examples of intracellular [Ca^2+^] traces showing the effects of SiNPs when applied at 1 μg/mL (**a**), 3 μg/mL (**b**) or 10 μg/mL (**c**), before and during the 2nd application of 10 μM ATP. The arrows point to the increase in intracellular [Ca^2+^] induced by the application of the SiNPs. (**d**) Ratio between the amplitudes of the responses to the 2nd and 3rd applications of ATP and the amplitude of the response to the 1st application of ATP, as a function of the concentration of SiNPs. The data are represented as mean ± s.e.m. (*n* = 135, 135, 52 and 92 for SiNPs at 1, 3, 10 and 100 μg/mL, respectively. The asterisks (**) denote statistically significant difference with respect to the corresponding control data [SiNPs] = 0 μg/mL (*p* < 0.01; Kolmogorov-Smirnov test).

To determine if SiNPs alter the responses to ATP, we used the same protocol described for Figure 1, but applying SiNPs 7 min prior and during the second stimulation with 10 μM ATP, all during perfusion of the standard Krebs solution (Figure 3a–c). Notably, SiNPs increased the baseline [Ca^2+^] prior to the second ATP application, in accordance with our previous study [30]. This increase in [Ca^2+^] appeared not to be reversible after washout of the nanoparticles for at least the next 9 min of duration of the experiment (Figure 3a–c). The 7 min pre-application of SiNPs was required to assess the effects of ATP without interfering with the increase in intracellular [Ca^2+^] elicited by the particles. SiNPs were applied at several concentrations (1, 3, 10, or 100 μg/mL). To quantify the effects of SiNPs, we determined the ratio between the amplitudes of the responses to the second or third application of ATP and the amplitude of the responses to the first application of ATP. SiNPs reduced the amplitude of responses to ATP in a concentration-dependent manner. The amplitude of the second response to ATP was not significantly affected during the application of 1 μg/mL SiNPs (Figure 3a,d), but was reduced at higher concentrations (Figure 3b–d). The inhibitory effect of SiNPs was more pronounced on the third ATP application, after the removal of the particles from the extracellular solution (Figure 3d).

Viability tests using fluorescence activated cell sorting with propidium iodide as a marker of dead cells showed only a marginal increase in the number of non-viable cells after 10 min exposure to 100 μg/mL SiNPs (3.2% above the control level; Figure S1).

### 2.4. Prolonged Inhibitory Effects of SiNPs on the Responses to ATP

**Figure 4:**
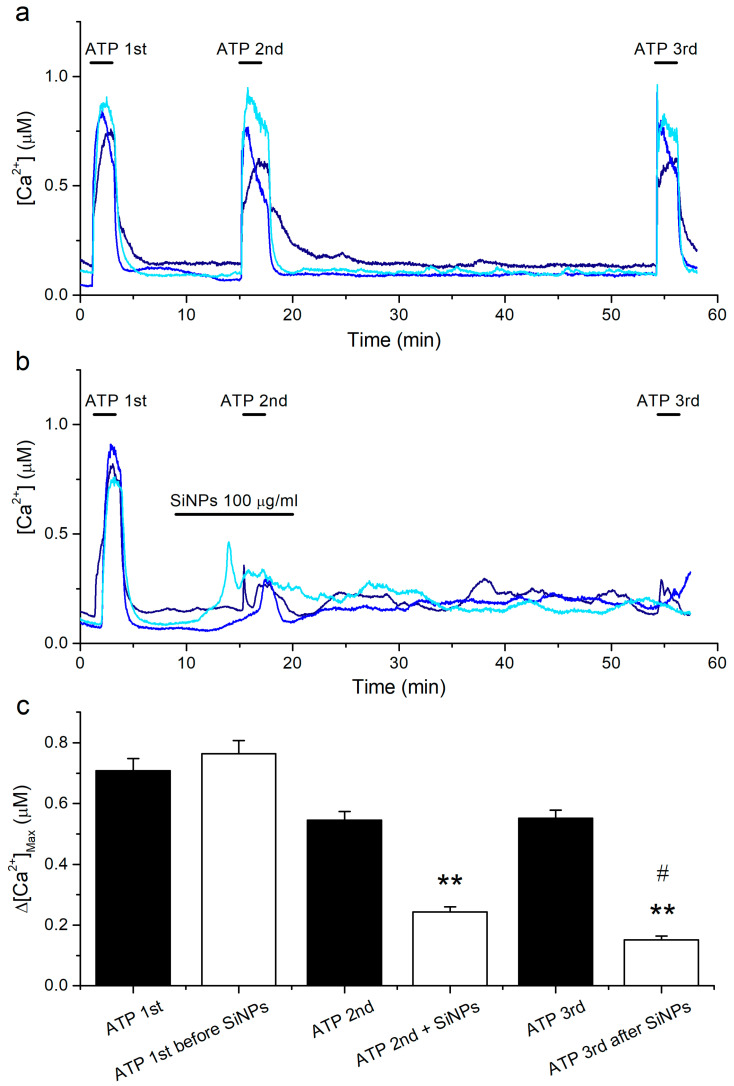
Persistent inhibitory action of SiNPs on responses to ATP. (**a**) Examples of intracellular [Ca^2+^] traces showing the effects of extracellular ATP applied at 10 μM. (**b**) Examples of intracellular [Ca^2+^] traces showing the effects of SiNPs when applied at 100 μg/mL, before and during a second application of 10 μM ATP. (**c**) Amplitude of the responses to three consecutive applications of ATP for the control series of experiments (black bars, *n* = 164) and for a series of experiments in which 100 μg/mL SiNPs was applied 9 min before and during the second application of ATP (white bars, *n* = 140). The data are represented as mean ± s.e.m. The asterisks (**) denote statistically significant difference of the data obtained in SiNPs with respect to the corresponding control data in the absence of SiNPs (*p* < 0.01; Kolmogorov-Smirnov test). The # symbol denotes statistically significant difference between ATP2nd + SiNPs and ATP3rd after SiNPs (*p* < 0.01; Kolmogorov-Smirnov test).

The above results show that SiNPs not only inhibit the response to ATP when they are applied simultaneously, but also affect the response to a subsequent ATP application 9 min apart. To determine if the reduction in ATP response caused by SiNPs persists after a longer time, we modified the previous protocol by delaying the third ATP application by 30 min. We found that the responses to a third application of ATP after almost 1 h of keeping the cells in Krebs solution were largely preserved (Figure 4a,c).

This allowed us to confidently assess the effect of SiNPs on a delayed exposure to ATP. Using this protocol, we observed that SiNPs application resulted in an increase in the intracellular [Ca^2+^] throughout the remaining measurements, i.e., long after SiNPs washout (Figure 4b). However, more critically to the current study, we found that the responses to ATP did not recover after 35 min of washout of the SiNPs (Figure 4b,c).

### 2.5. Effects of Intracellular Ca^2+^ Overload on the Responses to ATP

**Figure 5:**
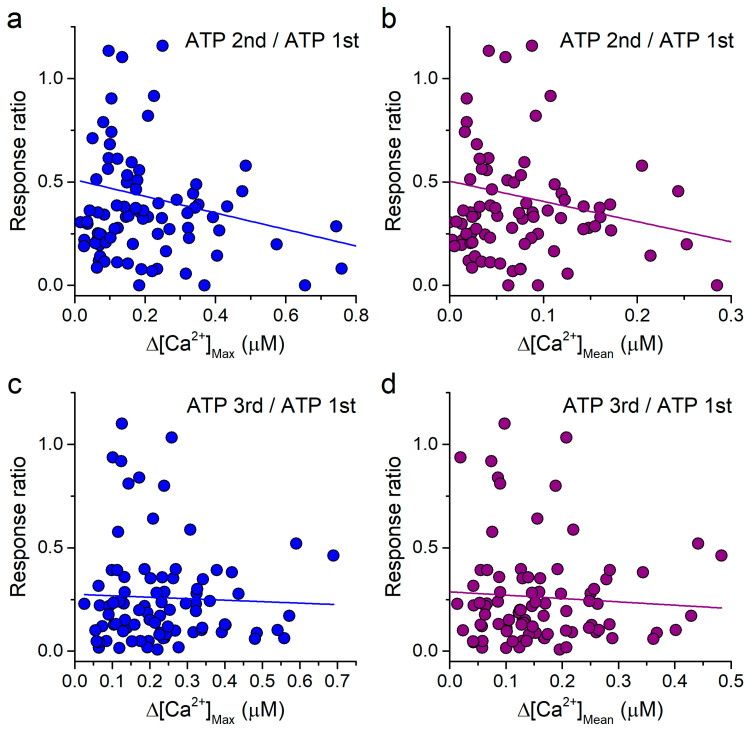
Lack of correlation between the reduction in the response to ATP and the increase in intracellular [Ca^2+^] induced by SiNPs. (**a**,**b**) Plots of the ratio of the amplitudes of the responses to the 2nd and 1st applications of 10 μM ATP versus the maximal increase (**a**) and mean increase (**b**) in intracellular [Ca^2+^] induced by 100 μg/mL SiNPs. (**c**,**d**) Equivalent plots to those shown in panels (**a**,**b**), but using the ratio of the amplitudes of the responses to the 3rd and 1st applications of 10 μM ATP. The lines represent the corresponding linear fits, with R^2^ values of 0.04, 0.04, 0.002 and 0.004, for panels (**a**–**d**), respectively.

SiNPs on their own induce an increase in [Ca^2+^] that, hypothetically, might lead to inhibition of the response to ATP. As a way to assess this possibility, we tested whether the inhibition of the response to ATP correlated with the increase in intracellular [Ca^2+^] induced by the previous application of 100 μg/mL SiNPs. For this, we used the data corresponding to the point 100 μg/mL SiNPs presented in Figure 3d. We found that the ratio of the amplitudes of the responses to the second and first applications of ATP, which is a direct measure of the effect of the SiNPs, did not correlate with either the maximal increase or the average increase induced by the application of SiNPs previous to the second ATP application (Figure 5a,b). The lack of correlation was even more obvious between the effects of the SiNPs on the third ATP application and the previous intracellular Ca^2+^ levels, i.e., maximal or mean values (Figure 5c,d).

**Figure 6:**
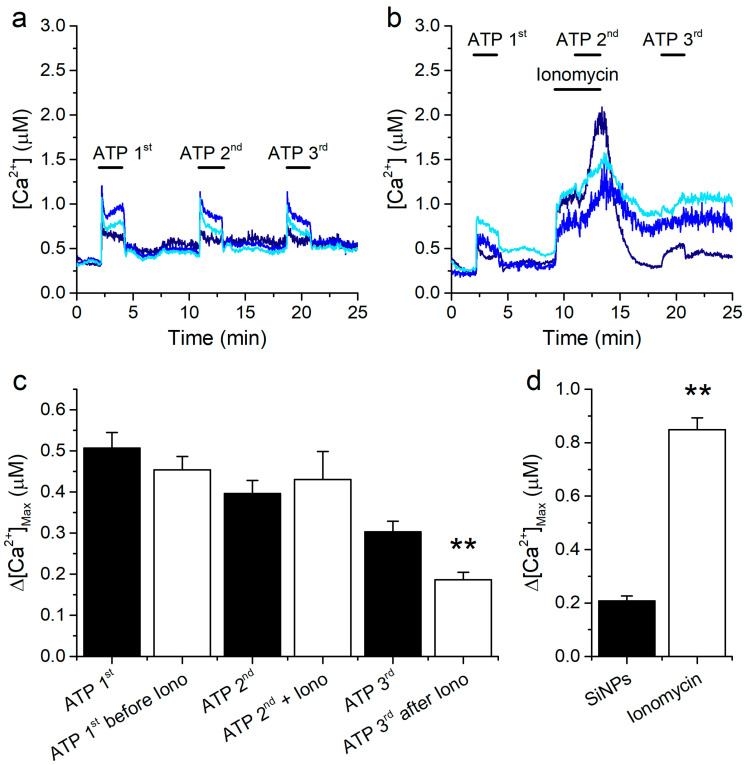
Response to ATP in 16HBE cells overloaded with Ca^2+^. (**a**) Example of control [Ca^2+^] traces recorded in 16HBE cells showing the responses to repetitive extracellular application of 10 μM ATP. (**b**) Examples of traces showing the effects of 1 μM ionomycin when applied before and during the second application of 10 μM ATP. (**c**) Amplitude of the responses to three consecutive applications of ATP for the control series of experiments (black bars, *n* = 36) and for a series of experiments in which 1 μM ionomycin was applied 2 min before and during the second application of ATP (white bars, *n* = 42). The data are represented as mean ± s.e.m. The asterisks denote statistically significant difference between ATP 3rd and ATP 3rd after ionomycin (*p* < 0.01; Kolmogorov-Smirnov test). (**d**) Comparison of the amplitude of the [Ca^2+^] responses induced by 100 μg/mL SiNPs and by 1 μM ionomycin (*n* = 92 and 42, respectively). The data are represented as mean ± s.e.m. The asterisks (**) denote statistically significant difference between the two bars (*p* < 0.01; Kolmogorov-Smirnov test).

We further probed for a relation between an increase in the intracellular [Ca^2+^] and the amplitude of the response to ATP by testing whether an increase in [Ca^2+^] induced by a mechanism distinct from that of the SiNPs has a similar inhibitory effect on the responses to ATP. We did this by perfusing the cells with the standard Krebs solution and testing the effects of adding the Ca^2+^ ionophore ionomycin (1 μM). A series of parallel control experiments showed that consecutive ATP applications triggered sizeable intracellular Ca^2+^ responses (Figure 6a,c; black bars).

As expected, ionomycin induced a fast and robust increase in [Ca^2+^], which was in fact much larger than that induced by SiNPs (Figure 6b,d). In contrast to the increase observed with the SiNPs, application of ATP during the perfusion of ionomycin did elicit an additional increase in [Ca^2+^] (Figure 6b). The amplitudes of these responses were not significantly different from the amplitudes observed in the control experiments (Figure 6c). We did observe, however, that the responses to ATP developed more slowly in the presence of ionomycin than in control condition (Figure 6b). This effect was not further characterized. The response to the third application of ATP appeared to be affected by the prior treatment with ionomycin (Figure 6b,c), but nevertheless this effect was smaller than that caused by 100 μg/mL SiNPs (see data for ATP 10 μM in Figure 2b).

### 2.6. Effects of SiNPs on the Concentration Dependence of the Response to ATP

**Figure 7:**
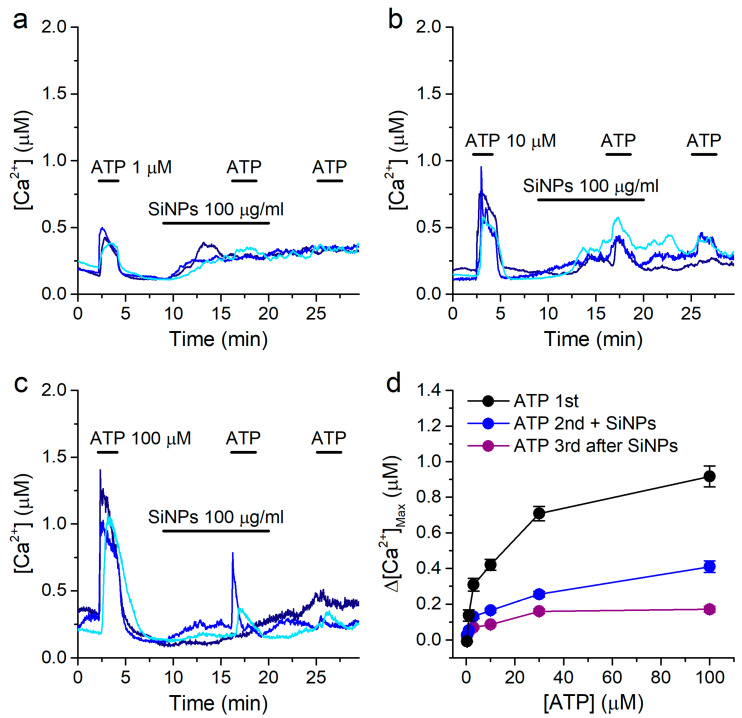
Effects of SiNPs on the concentration dependency of the response to ATP. (**a**–**c**) Examples of intracellular [Ca^2+^] traces showing the effects of 100 μg/mL SiNPs on the responses to extracellular ATP applied at 1 μM (**a**), 10 μM (**b**) or 100 μM (**c**). (**d**) Concentration dependence of the amplitude of the responses to ATP, for the 1st (control), 2nd (in the presence of SiNPs) and 3rd (after washout of SiNPs) applications. The data are represented as mean ± s.e.m. (*n* = 213, 68, 130, 109, 169 and 146 for ATP 0.3, 1, 3, 10, 30 and 100 μM, respectively).

In order to gain further insight into the mechanism of action of SiNPs, we determined how they alter the concentration dependency of the response to ATP. For this, we used the same ATP application protocol described above (Figure 1), but perfusing 100 μg/mL SiNPs 7 min prior and during the second stimulation with ATP (in the standard Krebs solution). We used SiNPs at this concentration because the effects were most robust both for the increase in intracellular [Ca^2+^] and for the inhibition of the responses to ATP, which was expected to result in less data variance. We confirmed the responses to ATP to be significantly inhibited in the presence of SiNPs (Figure 7a–d), and that the response to the third application of ATP was also inhibited, further demonstrating that SiNPs had an inhibitory effect even after they were removed from the extracellular solution (Figure 7a–d).

**Figure 8:**
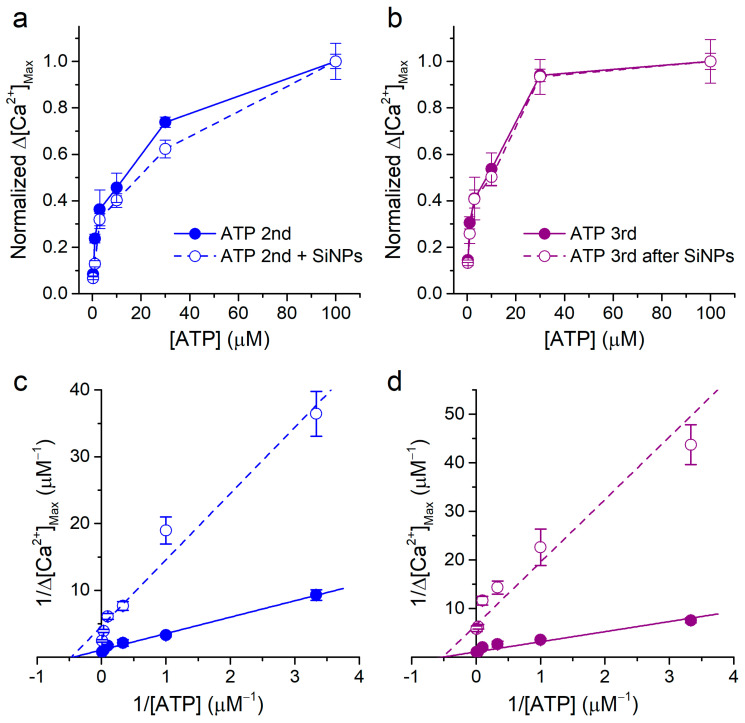
Non-competitive inhibitory effect of SiNPs on the response to ATP. (**a**,**b**) Normalized concentration dependence of the amplitude of the responses to ATP, for the 2nd application (control and in the presence of SiNPs) and for the 3rd application (control and after washout of SiNPs). The normalization was performed by dividing the data sets of Figure 1d and Figure 5d by the corresponding values obtained for 100 μM ATP. (**c**,**d**) Lineweaver–Burk plots of the data shown in Figure 1d and Figure 5d. The lines represent fits with linear functions. The data are represented as mean ± s.e.m. The n numbers are the same as in the original figures.

By comparing the data shown in Figure 7d with those in Figure 1d, it can be noticed that, apart from the obvious differences in maximal values, the shapes of the concentration dependencies determined in the absence and in the presence of 100 μg/mL SiNPs appeared to be similar. To test whether this was indeed the case, we replotted the data by normalizing each curve to the respective value obtained at ATP 100 μM (Figure 8a,b).

The comparison of the normalized curves revealed a large overlap of the data obtained in the presence and during washout of the SiNPs with the respective normalized control curves. Finally, we represented these data as Lineweaver–Burk plots (1/Δ[Ca^2+^] vs. 1/[ATP]) and fitted them with linear functions (Figure 8c,d). The fits yielded linear functions with slopes that were different between control (2.45 ± 0.11 and 2.08 ± 0.24 for second and third ATP applications, respectively) and SiNPs (9.92 ± 0.9 and 12.9 ± 3.3 for second and third ATP applications, respectively). In contrast, the resulting intercepts with the 1/[ATP] axis, which relate to the inverse of the apparent equilibrium constant (−1/K_M_), were nearly identical for control and SiNPs conditions (second ATP application: −0.44 ± 0.08 μM^−1^ and −0.47 ± 0.17 μM^−1^, respectively, and third ATP application: −0.52 ± 0.08 μM^−1^ and −0.52 ± 0.21 μM^−1^, respectively). The corresponding K_M_ values for the second ATP application were therefore 2.27 ± 0.4 μM and 2.2 ± 0.8 μM for control and SiNPs, respectively, and for the third ATP application, 1.9 ± 0.3 μM and 1.9 ± 0.8 μM for control and SiNPs, respectively. This analysis shows that the SiNPs only decreased the maximal response (efficacy) and not the sensitivity to ATP, suggesting that they have a non-competitive inhibitory action.

## 3. Discussion

Extracellular ATP is a key signaling molecule for the physiology of the airway epithelium, as it mediates responses such as the increase in ciliary beat frequency and mucus production and triggers the cough reflex. Moreover, ATP is a key messenger of cellular damage, and its detection plays a key role in activating protective mechanisms in neighboring healthy cells. Therefore, an insufficient ability to trigger a full response to ATP is expected to lead to reduced protective responses. Previous studies have established the implication of ATP-mediated signaling upon exposure to nanomaterials, including silica. However, these have mainly reported the induction of ATP release upon long-term exposure, from 30–40 min [47,48,49] to several hours [46,68,69]. In contrast, the acute effects of the nanoparticles on ATP-mediated signaling, that is, on the response to ATP itself, remained to be investigated. To cover this information gap, in this study we assessed the acute effects of 10 nm SiNPs on the responses of cultured human airway epithelial cells to ATP.

We used the 16HBE cell line, which has been referred to as an excellent model for studying ATP-induced intracellular Ca^2+^ transients in human airway epithelial cells [70]. Accordingly, we found these cells to respond consistently to extracellular application of ATP in the range between 0.3 and 100 μM. These responses had amplitudes that increased with the ATP concentration and displayed the typical morphology, with a fast upstroke phase followed by a biphasic decay [70]. We further found that cells responded to a first application of ATP with Ca^2+^ transients of normal amplitude in free extracellular Ca^2+^ solutions. This shows that in our experimental conditions, 16HBE cells respond to ATP via activation of P2Y receptors, leading to IP_3_-induced Ca^2+^ release from intracellular stores via type 3 IP_3_ receptors, as previously reported [70,71]. Interestingly, the second and third applications of ATP in Ca^2+^-free solution failed to evoke responses, which may have been due to a strong depletion of the intracellular stores induced by the first ATP application. In turn, this indicates the crucial importance of the replenishing mechanisms for the maintenance of responsiveness to repetitive stimulation with ATP in physiological conditions (in the presence of extracellular Ca^2+^). We indeed found that in the latter condition, consecutive applications of ATP several minutes apart induced reproducible responses. This allowed us to assess the effects of SiNPs by comparing the amplitudes of the responses to ATP in the absence and in the presence of SiNPs in the same cells.

Using this experimental paradigm, we found that SiNPs reduce the response to ATP in a concentration-dependent manner. This inhibitory effect was not only observed in the presence of the SiNPs, but also found to persist and to be actually stronger up to 35 min after the removal of SiNPs from the extracellular solution (Figure 3, Figure 4 and Figure 6). Future experiments may serve to determine whether the inhibition of the responses to ATP is reversible over longer periods of time. Nevertheless, the prolonged irreversibility of the inhibitory action of SiNPs demonstrates that the primary underlying mechanism is not mediated by SiNPs acting from the extracellular side of the membrane. Importantly, the viability tests showed only a very small increase in the number of non-viable cells following exposure to the highest SiNPs concentration tested (100 μg/mL). This result agrees with the stability of the intracellular [Ca^2+^] recordings in all series of experiments, indicating that no major changes in cell functionality leading to compromised plasma membrane integrity underlie the observed acute inhibitory effect of SiNPs on ATP response.

Another hypothesis to consider regarding the mechanism of action of the SiNPs is that, because of their negative surface charge, the nanoparticles might chelate intracellular cations and thereby decrease the intracellular Ca^2+^ signals detected with Fura2. However, this mechanism can be discarded, as it is expected to operate for all intracellular Ca^2+^ signals, and we have previously demonstrated that the SiNPs actually enhance the response of TRPV1 to the agonist capsaicin [30].

Alternatively, it could be envisaged that SiNPs inhibit the Ca^2+^ response by directly targeting components of the ATP signaling pathway. For instance, SiNPs may alter the mechanical properties of cellular membranes [38], and this could affect the function of ATP receptors. Furthermore, previous TEM studies have shown that SiNPs can be internalized via endocytosis through plasma membrane vesicles containing several nanoparticles and that this uptake process is accompanied by plasma membrane consumption and disruption [15]. This may lead to a decrease in the number of ATP receptors and to the formation of Ca^2+^-permeable pores in the plasma membrane, which in turn may explain the reduction of the responses to ATP and the SiNPs-induced increase in intracellular Ca^2+^], respectively.

However, the hypothesis considering that SiNPs directly target components of the ATP signaling pathway has a limitation. The cellular concentration of SiNPs and thereby any putative direct effect on a signaling component would be expected to decay, not to increase, upon washout. Thus, it remains difficult to explain why the concentration dependencies of the SiNPs action show a more potent effect on the ATP responses during washout than during the application of the particles.

A more plausible explanation is that the SiNPs trigger an inhibitory mechanism that outlives and is further enhanced after the ceasing of the accumulation of the nanoparticles in the cellular compartments. In this direction, the increase in intracellular Ca^2+^ emerged as a candidate factor to be involved, as we observed that SiNPs induced such effect in a rather irreversible manner, in agreement with our previous report [30]. However, we found that the inhibitory effects of the SiNPs on the ATP responses were not correlated to the increase in [Ca^2+^] induced by the particles. Furthermore, the experiments in which we used ionomycin revealed that cells undergoing intracellular Ca^2+^ overload were able to respond, albeit more slowly, to a first application of ATP. Taken together, our data strongly indicate that the increase in Ca^2+^ induced by the SiNPs is not the primary factor underlying the decrease in the responses to ATP.

Although our present experiments did not allow pinpointing a precise target of SiNPs action, they did let us conclude that SiNPs act via a non-competitive inhibitory mechanism. Thus, these particles may affect any of the elements of ATP signaling cascade that determine the maximal intracellular Ca^2+^ response, but do not act on any factors determining the sensitivity to ATP. For instance, SiNPs may decrease the number of ATP receptors available for activation at the plasma membrane and/or their maximal level of activation, but do not decrease their affinity for ATP. The latter is informative, as it indicates that SiNPs do not interfere with the binding and unbinding of ATP to and from P2Y receptors. Furthermore, because we assessed the responses to ATP from the amplitude of the Ca^2+^ increase, none of the events that determine the sensitivity to ATP may be affected. Future experiments, complemented by mathematical modeling, may help in discerning what elements of the pathway may be affected or not by the SiNPs.

Another curious result was that the inhibitory effect of SiNPs appeared to reach a plateau at concentrations above around 10 μg/mL, indicating that they are unable to inhibit the responses to ATP completely when applied at concentrations up to 100 μg/mL. Interestingly, we previously found a qualitatively similar effect for the inhibition of TRPV4 in 16HBE cells, but the plateau for maximal inhibition was reached at concentrations higher than around 300 μg/mL [30]. The difference in the concentrations for reaching the plateau phase of the two effects suggests that a concentration-dependent change in properties of the SiNPs is not the cause of this phenomenon. Alternatively, SiNPs might affect differently distinct pools of ATP receptors or other elements of this signaling pathway, or the particles might not have the ability to fully abrogate their maximal activation.

The effects of SiNPs must be undoubtedly linked to their physical and chemical properties. The specific contributions of each of these properties to the cellular actions of the particles could be studied by comparing the effects of particles differing only in one property—for instance, particles having exactly the same size, texture, etc., but different zeta potential. However, we do not have such particles at our disposal, and at this point we cannot make any inference on how specific particle properties determine their actions on the responses to ATP.

A key aspect of our findings is that the SiNPs concentrations required for the inhibition of the responses to ATP (1–3 μg/mL) are much lower than those needed to induce cytotoxicity or to observe cytokine release in vitro (25–6000 μg/mL) [72,73,74]. Furthermore, the time scale for the effects we reported here was between 3- to 150-fold shorter than those required for other reported SiNPs effects. This suggests that the ATP signaling pathway is a primary and very sensitive target of SiNPs. Finally, it should be noted that the inhibitory actions of SiNPs we reported here and previously on TRPV4 were specific, as the same particles enhanced the activation of the capsaicin receptor TRPV1 [30].

Our results demonstrate that SiNPs induce an acute non-competitive inhibition of the P2Y-mediated intracellular Ca^2+^ responses of cultured human airway epithelial cells to ATP. This effect occurs significantly faster and at concentrations lower than those previously reported for the induction of cellular toxicity and pro-inflammatory responses. Future research should be conducted to determine the molecular mechanisms of these actions, to test whether SiNPs are general inhibitors of purinergic signaling pathways in other cell types, and to determine whether SiNPs reduce the ability of airway epithelial cells to mount protective responses via the ATP signaling pathway, i.e., the increase in mucociliary clearance.

## 4. Materials and Methods

### 4.1. Ludox^®^ SiNPs

SiNPs used in this study were purchased from Sigma-Aldrich (Bornem, Belgium) as the commercial source of 30% wt suspension in water. Their basic properties were characterized in previous studies by our group [30] and can be found in Table 1.

For the experiments, the nanoparticle suspension was diluted to the desired concentrations in Krebs or Ca^2+^-free Krebs solutions (see below Reagents and Solutions).

### 4.2. Cell Culture

Human bronchial epithelial cells (16HBE) were grown in Dulbecco’s modified Eagle’s medium: nutrient mixture F-12 (DMEM/F-12) containing 5% (*v*/*v*) fetal calf serum (FCS), 2 mM L-glutamine, 2 U/mL penicillin and 2 mg/mL streptomycin at 37 °C in a humidity-controlled incubator with 5% CO_2_ and were seeded on 18 mm cover slips coated with 0.1 mg/mL poly-L-lysine.

### 4.3. Ratiometric Intracellular Ca^2+^ Imaging

Ca^2+^-imaging experiments were conducted with the ratiometric fluorescent dye Fura-2 AM ester (Biotium, Hayward, CA, USA) as an indicator for free intracellular calcium. Cells were incubated with 2 μL Fura-2 for 30 min at 37 °C. Solutions were applied using a multi-barrel perfusion system. The intracellular [Ca^2+^] was calculated from the ratio of fluorescence measured upon alternating illumination at 340 and 380 nm. Experiments were performed using an inverted microscope with MT-10 illumination system and the xCellence Pro software of the microscope (Olympus, Planegg, Germany). All measurements were performed at 35 °C. Fluorescence intensities were corrected for background signal, and intracellular Ca^2+^ concentrations were calculated as described previously [75]. Data were analyzed and displayed using Origin (OriginLab Corporation, Northampton, MA, USA).

### 4.4. Data and Statistical Analysis

To determine the amplitude of the response induced by the compounds of interest, we subtracted the baseline [Ca^2+^] prior to the application from the peak value reached during challenging (value denoted by Δ[Ca^2+^]). The baseline was calculated by determining the mean [Ca^2+^] during the last 20 s before the application of the compound. Data are given as ± standard error of the mean.

### 4.5. Reagents and Solutions

All chemicals were purchased from Sigma-Aldrich (Bornem, Belgium). The solutions containing ATP were prepared by adding the appropriate amounts of a 50 mM Na_2_ATP stock solution to the corresponding extracellular solution (standard Krebs or Ca^2+^-free Krebs). Ionomycin, an ionophore that triggers Ca^2+^ influx, was used to show that the effect observed during the application of SiNPs is not calcium-mediated and thus is a result of the interaction of SiNPs with subcellular structures. Ionomycin at 1 μM concentration was obtained by dilution of 2 mM stock in Krebs. Solutions used in measurements performed in the absence of extracellular Ca^2+^ were prepared with Krebs titrated to pH 7.4 with NaOH (Table 2).

## Figures and Tables

**Table 1:** Properties of SiNPs.

Property	Value
Size	10.2 nm (P_10_ = 8.1 nm and P_90_ = 11.8 nm)
Shape	Spherical
Solid structure	Amorphous
Zeta potential	−20 ± 3 mV
Dispersion	Monodispersed
Endotoxin content	<0.05 EU/mL

**Table 2:** Composition of the solutions used in the experiments.

Krebs	150 mM NaCl
	6 mM KCl
	1.5 mM CaCl_2_ × 2H_2_O
	1 mM MgCl × 6H_2_O
	10 mM glucose
	10 mM 4-(2-hydroxyethyl)-1-piperazineethanesulfonic acid (HEPES)
Ca^2+^-Free Krebs	150 mM NaCl
	6 mM KCl
	1 mM MgCl × 6H_2_O
	10 mM glucose
	10 mM HEPES
	10 mM ethylene glycol-bis(β-aminoethyl ether)-N,N,N′,N′-tetraacetic acid (EGTA)

## Data Availability

Data is contained within the article or Supplementary Materials.

## Supplementary Materials

The following are available online at https://www.mdpi.com/article/10.3390/ijms221810173/s1.
