# Supplementary material for: Silica Nanoparticles Inhibit Responses to ATP in Human Airway Epithelial 16HBE Cells
Source: Int J Mol Sci. 2021 Sep 21;22(18):10173. doi: 10.3390/ijms221810173 (PMC8467126; doi:10.3390/ijms221810173)
Supplement: Supplementary file 1 [file ijms-22-10173-s001.zip › ijms-1383369-supplementary.pdf]

Supplementary information

Silica nanoparticles inhibit responses to ATP in human airway epithelial 16HBE cells

Alina Milici, Alicia Sanchez and Karel Talavera

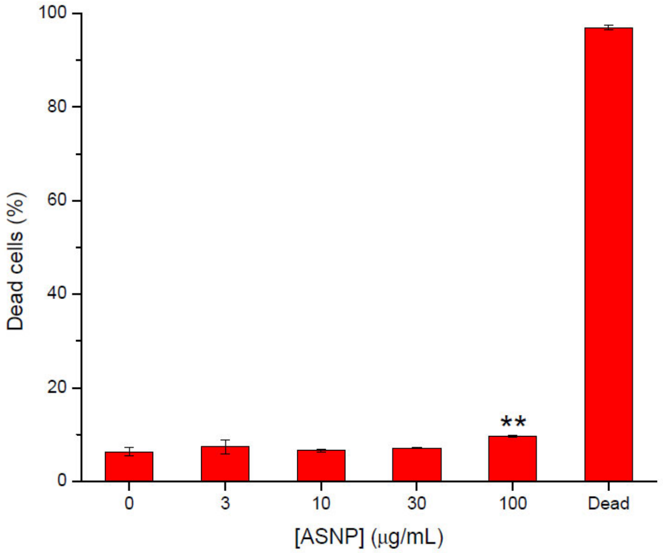

Figure S1. Viability test in 16HBE cells exposed to SiNPs. 16HBE cells were exposed to different concentrations of SiNPs for 10 min and a Fluorescence Activated Cell Sorting test was performed, using propidium iodide as a marker for detecting dead cells. After 10 min, cells exposed to 100 μg/ml SiNPs showed a significant decrease in viability compared to the population that was exposed to SiNPs (3.2% above the control level). However, none of the concentrations applied led to an increase in dead cell counts comparable to the positive control (a combination of ethanol and methanol).
